# Somalia's livestock–vector–human interface as a one health early-warning gap: Sentinel surveillance for MERS-CoV, Rift Valley fever, and CCHF

**DOI:** 10.1016/j.nmni.2026.101877

**Published:** 2026-10-27

**Authors:** Shukri Hassan Mohamed, Hibo Abdullahi Mohamed, Liban Abdullahi Sudi, Yakub Burhan Abdullahi

**Affiliations:** a Faculty of Health Sciences and Tropical Medicine, Somali National University, Mogadishu, Somalia; b Center for Health Research and Innovation, Somali National University, Mogadishu, Somalia; c Mogadishu Institute of Health, Mogadishu, Somalia; d Faculty of Health Sciences, Salaam university, Mogadishu, Somalia; e Benaadir Research, Consultancy & Evaluation Center (BRCE), Mogadishu, Somalia

**Keywords:** Somalia, Zoonotic surveillance, MERS-CoV, Rift valley fever, Crimean–Congo haemorrhagic fever

Dear Editor,

Somalia's pastoral and livestock-trade systems bring people into repeated contact with camels, small ruminants, and vectors through mobile herds, livestock markets, export quarantine stations, slaughter points, and animal-product handling. These interfaces create practical opportunities for early warning of zoonotic infections, particularly when animal, vector, and human health signals are monitored together. Rift Valley fever virus (RVFV) and Middle East respiratory syndrome coronavirus (MERS-CoV) were among the zoonoses prioritized for multisectoral One Health action in Somalia in 2023, whereas Crimean–Congo haemorrhagic fever virus (CCHFV) was not among the highest-ranked priorities [1]. We therefore propose surveillance for these three pathogens for different but complementary reasons: MERS-CoV and RVFV reflect recognized national priorities, while CCHFV is a more evidence-limited surveillance target whose inclusion warrants investigation at relevant livestock, tick, and occupational interfaces. Their proposed joint consideration is based not on equivalent national priority or shared ecology, but on the opportunity to use selected livestock-associated sentinel sites to detect pathogen-specific warning signals.

The strength of the evidence differed across the three infections. In northern Kenya, MERS-CoV RNA was detected in 83 of 243 (34.2%) prospectively followed camel calves, while three previously reported human PCR-positive cases clustered geographically and temporally with the camel outbreak, supporting probable sporadic spillover [2]. This study provides a regional example of how targeted camel surveillance can identify animal infections and relevant human warning signals. Somalia has documented serological evidence of RVFV exposure in ruminants, while communities along livestock-movement routes in Kenya had an RVFV IgG prevalence of 36.4%, with greater exposure among men and people reporting regular livestock contact or participation in animal birthing [3], [4]. Together, these findings support surveillance of livestock movement corridors. Contemporary in-country evidence for CCHFV remains limited; however, CCHFV RNA was detected in five *Hyalomma* tick pools collected from camels imported from Somalia to Egypt [5]. This supports CCHF as a surveillance hypothesis at relevant Somali livestock and vector interfaces but not as evidence of established contemporary human transmission in Somalia.

The rationale for considering these infections together is operational rather than ecological. MERS-CoV is primarily associated with dromedary camels, RVFV involves ruminants, mosquitoes, and direct contact with infected animal tissues, and CCHFV is principally maintained through tick–livestock cycles, with occupational exposure to infected ticks, blood, and tissues. Although these pathogens differ in their transmission patterns, their surveillance opportunities converge at a limited number of practical sentinel locations within Somalia's livestock system, including mobile herd routes, livestock markets, export quarantine stations, and slaughter facilities. These sites could capture complementary animal, vector, and occupational warning signals while retaining pathogen-specific triggers, sampling methods, and laboratory confirmation.

Reframing these interfaces as early warning platforms requires targeted sentinel and event-based surveillance rather than indiscriminate population screenings. First, selected high-volume livestock markets, export quarantine stations, and slaughter facilities can serve as sentinel sites. Second, surveillance signals should be pathogen-specific: camel respiratory sampling and investigation of compatible severe respiratory illness among highly exposed workers for MERS-CoV; livestock abortion or unusual mortality clusters, seasonal mosquito surveillance, and compatible acute febrile illness for RVFV; and *Hyalomma* tick surveillance together with investigation of unexplained febrile or hemorrhagic illness among slaughterhouse, veterinary, and other highly exposed workers for CCHFV. Third, surveillance intensity can increase following predefined triggers, such as abortion clusters, unusual livestock mortality, increased vector activity, or clusters of compatible human illnesses. Fourth, alerts, specimen referral, and laboratory confirmation should be fed into Somalia's Integrated Disease Surveillance and Response (IDSR) system and District Health Information Software 2 (DHIS2), alongside relevant animal health and One Health reporting mechanisms. Because entomological surveillance, molecular testing, and sequencing require specialized personnel and laboratory capacity, implementation should start at a small number of high-volume sentinel sites, use event-based triggers to prioritize testing, and strengthen specimen referral while the in-country diagnostic capacity develops.

Somalia's zoonotic preparedness should be judged not by whether every emerging infection is already documented nationally but by whether animal, vector, and occupational surveillance can detect and act on credible warning signals before zoonotic spillover emerges as an unexplained severe human disease.

## CRediT authorship contribution statement

**Shukri Hassan Mohamed:** Writing – review & editing, Writing – original draft, Conceptualization. **Hibo Abdullahi Mohamed:** Writing – review & editing. **Liban Abdullahi Sudi:** Supervision. **Yakub Burhan Abdullahi:** Visualization, Supervision.

## Consent to participate

Not applicable.

## Data availability statement

No new data were generated or analyzed for this Letter to the Editor. The findings discussed are based on previously published studies cited in the manuscript.

## Ethical approval

Ethical approval was not required for this study.

## Consent for publication

Not applicable.

## Availability of data and materials

No new data were generated or analyzed in this study. Therefore, data sharing is not applicable.

## Declaration of generative AI and AI-assisted technologies

ChatGPT (OpenAI) was used for language editing and formatting. The author reviewed the manuscript and takes full responsibility for its content.

## Sources of Funding

The authors received no financial support for the research, authorship, and/or publication of this article.

## Declaration of competing interest

The authors declare that they have no known competing financial interests or personal relationships that could have influenced the work reported in this study.
